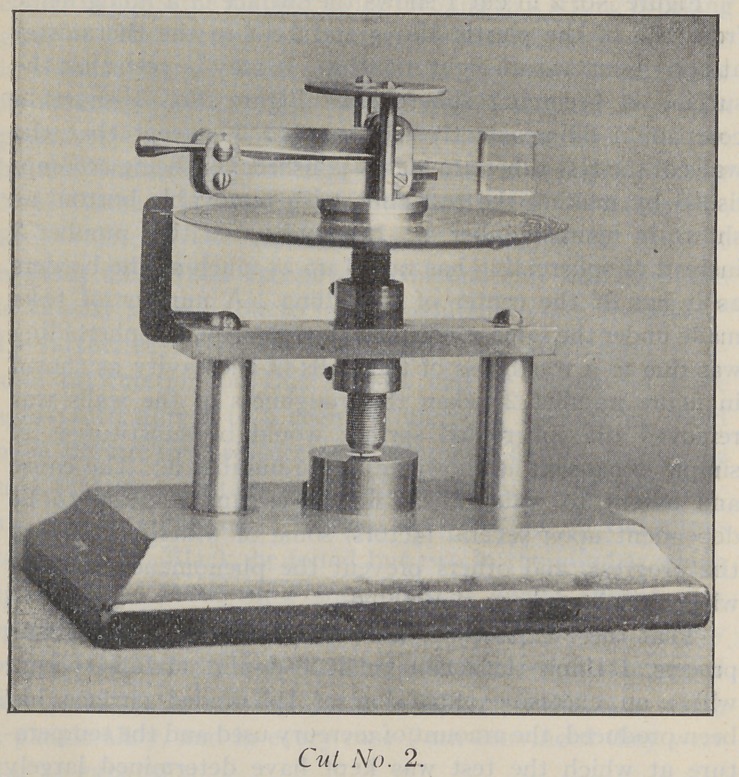# Amalgams and Their Peculiarities

**Published:** 1905-07-15

**Authors:** M. L. Ward


### AMALGAMS AND THEIR PECULIARITIES.

BY M. L. WARD, D.D.S.

Read before the Central Michigan Dental Association, March 28, 1905.

Nearly a century ago, amalgam was introduced to the dental profession as a remedy for rescuing decayed teeth from the ravages of a disease, which even now, in some countries, is supposed to be incurable and without adequate remedy. This remedy like many another has met with a cordial support by a part of the profession and an equally persistent warfare by another. It has enjoyed periods of success and other periods of almost universal condemnation. There are still sections of the country where these conditions have not materially changed, because of the use of poor alloys and careless manipulation, as may be seen by carefully reading the periodic literature of the current years. The impetus given the subject by its earliest advocates was renewed by the celebrated "new departure" enthusiasts who kept it constantly before the profession until Dr. Black took up a scientific investigation of the subject in 1895–6. He proved beyond a doubt that most of the alloys made at that time were in the range of greatest shrinkage, and that they contained large quantities of copper and tin which, in addition to the shrinkage, were largely responsible for the extreme discoloration of tooth structure that accompanied the use of alloys. It should be remembered that the history of the so-called hard alloys dates no further back than Dr. Black's work. Articles appear in our journals as late as January of 1905 with quotations from Taft's operative dentistry, and from Dr. A. J. Bennett's article in the *American System of Dentistry,* to show that gold is superior to amalgam as a filling material, while nothing definite was known about amalgam, and no one had even approached definite results until Dr. Black did his work in 1895–6. These two men were prominent in the profession and very proficient in other lines of work, but they knew nothing of amalgams alloys scientifically. Why should their work be used as references at this late day? The article referred to further says: "Still we have little evidence that the alloys now in use are very greatly superior as tooth preservers to those of many years ago, which were so objectionable on account of discoloring the teeth." It would be a good idea for such writers to begin with the new epoch in the history of amalgam, and study it carefully before making such statements about a material admitably inferior to gold, but certain to occupy a prominent place among our filling materials. As a result of Dr. Black's work, the alloys furnished us to-day are divided into two classes. There are comparatively few made now that do not fall into one of these classes. It is far from the findings of Dr. Black that this is true. There are a few manufacturers making them as he would have them. The best and first-class alloys are the hard alloys, composed of from 65 percent to 68 percent of silver, 26 percent to 28 percent of tin, 3 percent to 4½ percent of copper and 1 percent to 2½ percent of zinc. All of the best hard alloys fall within these ranges, but are not made from any one formula, a fact, which if thoroughly understood by the profession, would retard the progress, and in time obviate the use of alloys made from some "pet formula" by some silver-smith or other person equally as limited in his knowledge of the subject.

From this class rapid, medium and slow setting alloys are made, though the slow ones are much faster than the other class or plastic alloys. These hard alloys are sufficiently plastic when properly annealed. The small amount of movement accompanying their amalgamation and setting is all expansion. They give up their excess of mercury comparatively easy, and their color is good. They are the only ones that are stiff enough to remain where they are packed and not change their position under subsequent manipulations necessary to complete the filling. Their flow is comparatively little. Their resistance to crushing stress is great. Close observation and common honesty should have been sufficient to enable the profession to eliminate from the market entirely everything but the best of this class of alloys long before this. Instead of mastering the one and, apparently the only one, objection—viz—the hard working property, many of the profession cater to their personal likings and, regardless of the magnitude of the error, demand the soft or plastic alloys to which we will refer briefly for comparison. The composition of these alloys is based upon the short shrinkage and expansion range of fifty parts silver and fifty parts tin. The silver in these alloys ranges from 43 percent to 48 percent, the tin from 48 percent to 58 percent and the zinc from 1 percent to 2 percent. Most of these alloys are possessed of the dual movement, viz, both shrinkage and expansion. It must not be understood that this shrinkage and expansion are both going on at the same time. The shrinkage occurs first and usually within twenty-four hours, while the expansion does not begin till the second or third day. Even though the shrinkage which occurs during the first twenty-four hours is all made up by a subsequent expansion, but the borders are never where they were before these movements took place. These alloys will retain great excesses of mercury because of their stickiness, which excess, at body temperature, results in a continued crystallization lasting for months and perhaps years. Their flow is from five to ten times that of the hard alloys, which enables the continued crystallization to produce the protruding borders so often seen in fillings, especially in the four-walled cavities such as are found on the buccal surfaces of the teeth, the typically spheroided fillings which will be referred to later. Their resistance to crushing stress is very low. They cannot be packed by any means so as to make a good filling. Any effort to condense one portion of a filling results in a movement in some other part because of the softness of the mass. They are all very slow setting alloys. Rapid and medium setting alloys cannot be made from this class. The quick setting properties diminish quite rapidly as we leave the ranges given for the hard alloys, and it is almost lost before the alloys of this class is reached. As a result we have a mass that requires much time to stiffen sufficiently to admit the manipulation necessary, to complete the filling without disturbing it enough to spoil its adaptation. Conscientious and conservative practitioners will not long insert this class of alloys when they understand that with the same effort on their part and with no greater expenditure of money, and with the same ease to their patients, they can save more teeth and have better looking fillings. The great trouble is, and has been for years, that the properties of amalgam alloys have not been comprehended by the average practitioner. It has been necessary for them to accept the articles in our periodic literature, many of which are similar to the one I have noticed above. Probably not 5 percent of the profession understand that silver is an expanding constituent, and that because of its affinity for mercury and crystalline form it controlls the setting; or that it improves the color and increases the edge strength; or that it lessens the flow, and because of its great tendency toward crystallization and its property of uniting with mercury slowly at ordinary temperatures, it causes the alloy to work hard. They do not realize that tin, which unites with mercury in all proportions and at all temperatures, forms a weak crystalline compound, if crystalline at all, that it produces shrinkage, retards setting, reduces the edge strength and increases the flow. It is used simply because it imparts plasticity through a weak or non-crystalling form. Copper unites with mercury with difficulty, tarnishes readily with moist air and sulphuretted hydrogen, but it diminishes shrinkage, hastens setting, increases the edge strength and lessens the flow. Zinc unites with mercury in atomic proportions, tarnishes slowly, controls shrinkage markedly, hastens the setting, improves the color and edge strength and lessens the flow.

If the profession understood that these properties of each constituent control markedly the alloys, as is usually the case with solutions or mechanical mixtures, they would no longer use alloys made with less than 65 percent of silver in them. It is this lack of general understanding caused by the secrecy on the part of those in possession of the knowledge of the properties of alloys, that keeps a large percentage of the profession demanding these soft plastic alloys which the manufacturers know very well, do not compare with their hard alloys composed of higher percentages of silver. What can we expect them to do? They are simply supplying the demands of an uneducated public.

The hard alloys as furnished us to-day may be classified as rapid, medium and slow setting. The difference in these three classes being due to the manner and extent of annealing, their being little or no difference in composition. Regardless of the claims of some of the manufacturers that all of their alloys are carefully annealed and the movement accompanying their amalgamation and setting does not exceed one ten-thousandth of an inch expansion, it can be shown that this is true only in the earlier stages or crystallization or with the slow setting alloys. If these alloys are subjected to a test of six months or longer at body temperature, it will be seen that there is a marked difference between a rapid, medium and slow setting alloy in the amount of expansion that takes place. It will also be noticed that at the time of amalgamation there is a marked difference in the amount of mercury required to make a plastic mass of each. For this testing, I have two thermostats running all the time, one set at room temperature, one at body temperature, and no test is regarded as final that has not been in both of these from fifty days to six months. And often at the end of this time changes are found to be going on. For illustration I will give you the final results of some of the tests upon one of the hard alloys that was kept seventy-five days in the thermostat at twenty-one degrees Centigrade. The alloy was cut on a lathe with an automatic micrometer adjusting feed so that I probably had as uniform a cut as could be obtained. The test tubes were five-sixteenths of an inch in diameter and five-sixteenth of an inch high. The alloy was made in an electric furnace which is supplied with conveniences for preventing oxidation. The stirring and casting were done under the best obtainable circumstances and in accordance with methods advocated by expert metallurgists. There was little or no chance for error in the preparation of this ingot. The mixing and packing were done as I have described in another part of this paper, except that the mercury was added from a small jug which had been previously weighed.

Formula, Ag, 67.75; Sn, 27.50; Cu, 3.40; Zn, 1.35.

| No. | Annealed | Mercury | Expansion | Setting |
|---|---|---|---|---|
| 1 | None | 60.00 percent | 23 points | Extremely rapid |
| 2 | 1 day | 55.50 percent | 7½ points | Slower than No. 1 but quite rapid |
| 3 | 2 days | 53.41 percent | 4 points | Slower |
| 4 | 3 days | 53.00 percent | 2 points | Slower |
| 5 | 6 days | 52.89 percent | 1¾ points | Slower |
| 6 | 10 days | 51.76 percent | 1 point | Quite slow, but faster than the plastic alloys |

First, let us notice that the movement accompanying the amalgamation and setting of this alloy, as is always true with the best of these alloys, was all expansion. Shrinkage is not a phenomenon accompanying the use of the best of this class of alloys, if they are properly manipulated. The most careless manipulator among us probably could not make one of them shrink, though it is possible that a slight shrinkage might be produced by mixing too thin. Second, let us notice that this expansion is practically all removed by the annealing, thus producing a material whose movement is reduced to a minimum, not exceeding in the earlier stages of crystallization at ordinary temperature one ten-thousanth to two ten-thousandth of an inch on a block of amalgam five-sixteenth of an inch in diameter and five-sixteenth of an inch in depth. Third, let us notice that the rapid setting properties are lost as the annealing is continued. I do not mean to infer that the quick setting properties are all lost by the annealing, but that the extremely rapid setting tendency is reduced. When these alloys are fresh cut, it is almost impossible to make a plastic mass of them, though as much as 60 percent of mercury is used, they are still dry and granular, and stiffen the very moment they are undisturbed. With each day at room temperature and each hour at higher temperature, the quick setting properties are lost until we have an alloy that sets quite slowly, though not as slow as the plastic alloys. Let us bear in mind, then, that the difference in the annealing is what produces the difference in the setting properties, and that this annealing occurs at room temperature as well as at higher ones, only it takes a longer time. If, then, you are buying rapid setting alloys in large quantities, you should not be surprised to find the last bottle setting much slower than the first, unless you have kept it in a very cool place. Much stress is laid upon the proper amount of annealing by some of the manufacturers. By this they claim to get an alloy that always gives an expansion not exceeding one ten-thousandth of an inch on their rapid, medium and slow setting alloys. If you will refer to the table above, you would find that test No. 2 would have compared quite favorably to some of our hard alloys marked "Rather rapid setting," etc., and No. 5 just as favorably to one of our hard alloys marked "Slower setting," though there is quite a difference between the two in the amount of expansion that took place and the percentage of mercury required to make a plastic mass. Again, let me remind you that these good, hard alloys do not shrink. It is a question of how much expansion takes place and how quickly they set.

Let me show you the results of time on some other alloys. Some time ago I asked what alloys a certain dealer handled and which ones he sold the most of. He responded with the usual reply that he sold one hard alloy and one plastic alloy, but kept in stock a large number of others. The ones he kept in stock had been in his posession a long time. The ones he sold the most of were fresh, because his supply was being constantly exhausted and as often replenished. An analysis of the alloys in his stock seemed to cover the field fairly well, as will be seen by the following table:

| Alloy No. | Ag. | Sn. | Cu. | Zn. | Gold | Expansion | Average Shrinkage |
|---|---|---|---|---|---|---|---|
| 1 | 43.21 | 56.68 | ........ | .09 | ........ | ........ | 10 points |
| 2 | 45.64 | 53.14 | .15 | 1.10 | ........ | ........ | 2¾ points |
| 3 | 46.85 | 52.17 | ........ | .98 | ........ | ........ | 8¼ points |
| 4 | 48.61 | 50.04 | ........ | 1.55 | ........ | 2 | |
| 5 | 43.44 | 50.51 | 3.41 | 2.64 | ........ | ........ | ½ point |
| 6 | 54.08 | 39.92 | 2.56 | ........ | ........ | ........ | ........ |
| 7 | 58.07 | 34.93 | 3.46 | 2.54 | ........ | ........ | ½ point |
| 8 | 66.19 | 27.23 | 4.12 | 2.46 | ........ | 1½ | ........ |

Let us notice that they all shrank from one-half to ten points on the micrometer, except Nos. 4, 6 and 8. Nos. 4 and 8 were the ones he handled the most of. They were both fresh. No. 6 we happened to catch at zero point. These same alloys were now placed in a thermostat which was set at room temperature (thirty-eight degrees Centigrade, and left there six months. When these tests were repeated. At this time Nos. 4 and 6 had gradually crept into the shrinkage column, leaving No. 8 the only one to give the slight expansion necessary to insure against shrinkage. Nos. 4, 5 and 6 were not capable of great shrinkages, and it is barely possible that these slight shrinkages were due to the manipulation, but it will be seen that the percentage of silver in all three of them was below 55 percent, a fact, which alone is sufficient to suspect or condemn them. This investigation of this dealer's stock shows that the low grade alloys are still made and that many of the profession are using them.

The amalgamation of alloys is best accomplished with weighed proportions of mercury and alloy. It may appear a feeble, and prove a fruitless effort for me to advocate what Dr. Black has advocated ten years, but it is true nevertheless, that this is the only way to get uniform results. These proportions will vary with the alloy used, and perhaps the age of some alloys, as has just been shown. The amount of mercury that should be used with these alloys is generally greater than the alloy, ranging in most cases from four parts alloy to five of mercury, to five parts of alloy and seven of mercury. After weighing the alloy and mercury they should be placed in a small mortar and stirred rapidly but lightly with a pestle until the two are coherent enough to be turned into the land conveniently. Much force should not be used in the mortar, lest the identity of the cut be entirely destroyed. This should result in incorporating the two without loss of material or waste of time, and without comminuting the alloy. I am satisfied that the alloy may be much more uniformly dissolved in the mercury by a continued, rapid kneading in the hand, than by grinding in a mortar. After some careful study of this subject, I found that unless the grinding process, as done with the mortar and pestle, was carried on for a long time and much care used in keeping the borders well shaken into the middle of the mortar, a section would show patches of what appeared to be a complete solution of the alloy in the mercury, among which were granules of the alloy that had not been dissolved. After incorporating the alloy and the mercury in the mortar, they should be turned into the hand and kneaded rapidly until the granulations have quite uniformly disappeared. If, during the kneading, the mass becomes real soft, before the granulations disappear, the surplus mercury should be removed by gently squeezing the mass between the thumb and finger. Do not use much force because you will press too much mercury from the center into the border of the mass. Do not take the time to place the mass into a chamois skin or muslin, unless you have a mass too large to be uniformly compressed between the thumb and finger, because the alloy will stiffen so much that subsequent kneading will not produce the required plasticity. After removing the excess of mercury, the mass should again be returned to the hand and kneaded vigorously. If the mass again becomes too soft, more mercury should be removed as before. It is usually necessary to return the mass to the hand three or four times, each time removing a *little* mercury, to get the "creaking noise" which indicates the amount of stiffness necessary to enable it to be properly packed. It must be understood that "weighed proportions" of mercury and alloy does not mean the amount of mercury which should be left in the filling, but the amount which should be used to amalgamate the alloy. It is the amount of mercury which, after being incorporated with the alloy in the mortar and thoroughly kneaded in the hand, will allow us by good firm pressure between the thumb and finger to remove a very small globule of mercury, and after more vigorous kneading in the hand, will allow little or no mercury to be removed with the same pressure as was used the first time. Those who are not using weighed proportions of mercury and alloy should use great care not to add mercury from a jug till large globules of mercury can be removed with little effort. As soon as enough mercury has been added to make the mass sufficiently coherent to hold together the kneading should be begun and mercury added very slowly until a point is reached where, after vigorous kneading, a very small globule of mercury only may be removed. This is the best way to determine the proper proportions and should be observed closely, for the reasons which will be given later under the head of constituents removed with excess of mercury. The packing should be begun at once, using as much force as is consistent with the operation, and as large, flat-ended instruments as will conveniently enter the orifice of the cavity. If the cavity contains angles or irregularities, as large an instrument should be used as will compress the amalgam into such an angle. Dr. Black says the whole principle of making perfect work is contained in the one word compression. This class of alloys all have a tendency to stiffen if they lie still for a few seconds. It is this property that enables us to pack them so much better than the plastic alloys. After the amalgam has once been carried to place, it should not be moved again under any circumstances. Any subsequent movement of the mass will produce a weak filling. The amalgam should lie still after it has been packed. The use of the matrix is invaluable and almost imperative. It is impossible to get any degree of compression in a cavity that does not contain four natural or artificial walls. There is no one thing that will be so edifying as to study carefully the borders of your amalgam fillings with even a low power lens. Expert manipulation, attended by the most discriminating care, will not produce a border on an amalgam filling that is as good as the border of an ordinary gold filling that has been made with anything like favorable conditions. An amalgam filling which has been made without a matrix and under other less favorable circumstances is not deserving of comparison. With amalgam, as with other plastic fillings, the cavity should be filled more than full and left so until it has stiffened sufficiently to admit the necessary carving without movement in the mass. The finishing should be the same as with a gold filling and should be done every three or four months because of the expansion that is always liable to go on with our present alloys, unless care has been used in selection of the alloy, and the proper percentage of mercury used to amalgamate it. An understanding of the merits and manipulation of the hard alloys should be general. The use o the so-called gold and platinum alloys, the plastic alloys, Dr. So and So's special alloy, the used of the round burnisher for packing, filling proximal cavities without a matrix, the washing of alloys, the discoloration of tooth structure, and the manufacture of one's own alloy should be matters of history, and no longer material for discussion at dental meetings. If I could prevent the sale of the plastic alloys and hinder dentists from making their own alloys and stimulate the thousands who have labored for years with indifference and inattentiveness to this subject of manipulation, I should feel that I had exploited the most fertile field in dentistry, and would reap a harvest that had been ripe for years. There is a wide spread belief that the composition of a given alloy may be markedly changed by the excess of mercury necessary to amalgamate it. Such, however, is not the case with the best alloys when they are *properly* manipulated. There is a change produced, but it is not a perceptible one in ordinary work.

I recently made a study of this subject to determine whether or not the constituents carried out with the excess of mercury were in the same proportion as they were in the alloy. The tests and the results on one or two of these alloys will be sufficient to illustrate what takes place. I purchased in open market one of the hard alloys that was very uniformly cut and had given good results when tested in the laboratory. It analyzed silver 65.59 percent, tin 27.67 percent, copper 4.39 percent and zinc 2.37 percent. I saved the expressed mercury from fifteen fillings that I had inserted (while using this alloy in my practice). These fillings were manipulated as I have previously described, using 47.25 percent of alloy and 52.75 percent of mercury. The excess of mercury was removed by gentle pressure between the thumb and finger. An analysis of this showed the silver, copper and zinc to be within .01 percent what they found in the alloy, and the tin to be in excess of what it was in the alloy, .32 percent. From this same alloy I made seven fillings in the laboratory, using 55 percent of mercury. Each one was stirred rapidly in a mortar for one minute and kneaded vigorously in my hand for four minutes. You will notice that this was too much mercury and it was kneaded entirely too long with the excess of mercury, making it sloppy. But I was making a special effort to dissolve greater proportions of the alloy this time if possible. The excess of mercury this time was removed through a strong, fine piece of muslin, using about all the force I could exert. An analysis of this showed the silver, copper and zinc to be within .2 percent of what they were in the alloy, and the tin to be in *excess* of what it was in the alloy, 1.01 percent.

Still another test was made upon this same alloy with the conditions exactly the same, except that the excess of mercury was removed by heavy pressure between the thumb and finger. This time I removed larger percentages of all the constituents.

The tin this time was in excess 3 percent and the silver lacking about the same amount, while the copper and zinc remained almost the same.

Another alloy that analyzed silver 64.39 percent, tin 27.54 percent, copper 4.47 percent and zinc 4.60 percent, was treated axactly the same as the one just described and gave practically the same results, viz, the silver was lacking 2.67 percent, the tin in excess 2 percent, the zinc was in excess .34 percent and the copper about the same.

With these as with my other tests the proportion of tin that was removed with the excess of mercury has always been greater than it was in the alloy—the proportion of silver has always been less—the copper has always nearly equalled to, but never exceeded—and the zinc has always equalled to, and often exceeded.

These analyses indicate to a certain extent the selective power of mercury for each constituent, and show that with large percentages of mercury and excessive kneading with this percent of mercury, we may produce marked changes in our alloy. We would naturally think that since tin is regarded as the shrinking constituent, that the removal of a greater or less amount of it would increase the expansion of the alloy. Such, however, is not the case. Shrinkage accompanies the removal of this tin every time. I have not a single exception. This is the reason why we should work closer when we are amalgamating our alloys and not have large amounts of mercury to remove.

The so-called spheroidal tendency of amalgams has always occupied a sufficiently conspicuous place among the phenomena accompanying the use of amalgam, and been given more or less lengthy discussions in our text-books and journals, and attracts some attention even to-day.

The prevailing opinion seems to have been, and is now, that the spheroidal shape of globules of mercury were due to some property peculiar only to mercury and this property of the mercury was directly responsible for the spheroided amalgams. We have lost sight of the fact that other metals too are spherical when in a liquid state, and that the liquid state of mercury is simply a matter of temperature. Of the articles now on record, I will refer to but two that seem to have any bearing upon the subject as it presents itself to me, after a somewhat extended study. In the*Dental Cosmos*, Vol. 37, page 569, Dr. Black says that spheroided fillings may be due to an expansion of the mass, being confined by the cavity walls, the material will rise up in the center in the same manner as ice forming in a strong drinking glass or other vessel will assume a spherical surface. This is because the ice flows under stress the same as amalgam flows under stress. "The second article may be found on pages nine and ten of a complimentary pamphlet sent out by the Garhart Dental Mfg. Co., which is a paper read by N. K. Garhart, before the Odontological Society of Louisville, Ky., He says: "Spheroiding or changing of form is nothing more than excessive shrinkage, and this condition is directly attributable to faulty manipulation of the alloy." He further says that Dr. Black could have given some valuable information on this subject had he submitted some spheroided fillings to a chemical analysis, because he could have proved the presence of surplus mercury." Notice, he says that they contain an excess of mercury, and that they are due to shrinkage. Hodgen says that of all the alloys tested by Dr. Black, he found but two in the number which spheroided. From these statements it would appear that spheroiding is very rare and that there is still a variety of opinions as to its cause. I have seen scores of spheroided fillings in the mouths of patients whose dentist was using a plastic alloy, and in every case I am convinced they were due to expansion.

*Cut No.* 1.

Figure No. 2 in cut 1 shows the surface of a filling made from one of the plastic alloys and kept in the thermostat at body temperature eight months. It may be seen that the surface is becoming spheroided. Figure No. 3 shows a companion filling exactly like number 2, except that the walls of the test tubes are highly polished, this being accomplished by making the test tube with removable bottom as shown in figure number 4. It may be seen that number 3 instead of spheroiding has raised up as much at the borders as it has at the center of the filling. A number of tests made under the same conditions have shown that spheroiding was due to a roughness of the walls of the cavity as shown in figure number 2, when this roughness of the walls was removed the spheroided surface would be substituted by simple expansion as shown in figure number 3. The cause and extent to which these fillings expand appears to be dependent upon several factors, some of which will retard the progress, and others prevent the phenomenon entirely when they have been eliminated.

That these expansions are a continuation of the setting process, I think there can be little doubt. In every case where an excessive expansion or spheroided surface has been produced, the amount of mercury used and the temperature at which the test was kept have determined largely the amount of expansion obtained. These expansions are not obtained from all grades of alloys. Only those that retain great excesses of mercury and have a large percentage of the non-crystalline element produce them. This class I have already designated as the plastic alloys, composed of silver, tin, and zinc, in the proportions previously given. From the evidence I now have I think I am safe in stating that these expansions may be attributed to the property of these alloys to flow easily under the strain of crystallization due to loosely or uncombined mercury in the tin, acting with the aid of body temperature, upon a partially crystallized alloy.

*Cut No. 2.*

Cut No. 2 shows an amalgam micrometer which may be used in connection with the Wedelstadt test tubes or test tubes open at both, top and bottom. It consists of a micrometer screw which runs through a double sectional nut, a disk containing the graduations which are read at the vertical bar and two levers which swing in the front of a mirror and indicate when a contact has been made with the filling by means of the needle which runs through the micrometer screw. Bearings for levers are jeweled and nut for screw is adjustable.

## Figures and Tables

**Cut No. 1. f1:**
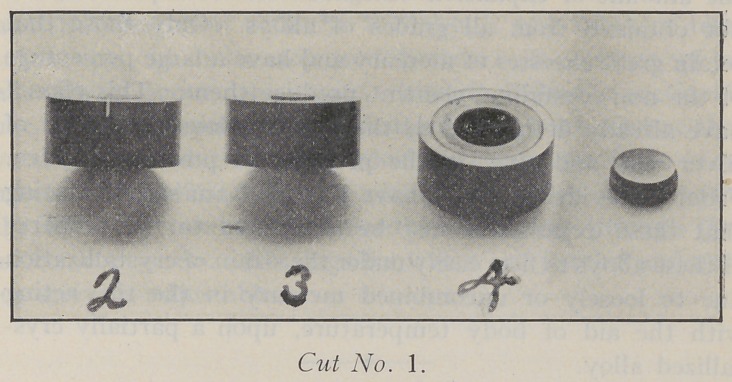


**Cut No. 2. f2:**